# The crystal structure of human Rogdi provides insight into the causes of Kohlschutter-Tönz Syndrome

**DOI:** 10.1038/s41598-017-04120-x

**Published:** 2017-06-21

**Authors:** Hakbong Lee, Hanbin Jeong, Joonho Choe, Youngsoo Jun, Chunghun Lim, Changwook Lee

**Affiliations:** 1 Department of Biological Sciences, School of Life Sciences, Ulsan National Institute of Science and Technology, 50 UNIST-gil, Ulsan, 44919 Republic of Korea; 2 Department of Biological Sciences, Korea Advanced Institute of Science and Technology, Daejeon, 34141 Republic of Korea; 3 School of Life Sciences, Gwangju Institute of Science and Technology, Gwangju, 61005 Republic of Korea; 4 Cell Logistics Research Center, Gwangju Institute of Science and Technology, Gwangju, 61005 Republic of Korea

## Abstract

Kohlschutter-Tönz syndrome (KTS) is a rare autosomal-recessive disorder of childhood onset characterized by global developmental delay, spasticity, epilepsy, and amelogenesis imperfecta. Rogdi, an essential protein, is highly conserved across metazoans, and mutations in Rogdi are linked to KTS. However, how certain mutations in Rogdi abolish its physiological functions and cause KTS is not known. In this study, we determined the crystal structure of human Rogdi protein at atomic resolution. Rogdi forms a novel elongated curved structure comprising the α domain, a leucine-zipper-like four-helix bundle, and a characteristic β-sheet domain. Within the α domain, the N-terminal H1 helix (residues 19–45) pairs with the C-terminal H6 helix (residues 252–287) in an antiparallel manner, indicating that the integrity of the four-helix bundle requires both N- and C-terminal residues. The crystal structure, in conjunction with biochemical data, indicates that the α domain might undergo a conformational change and provide a structural platform for protein–protein interactions. Disruption of the four-helix bundle by mutation results in significant destabilization of the structure. This study provides structural insights into how certain mutations in Rogdi affect its structure and cause KTS, which has important implications for the development of pharmaceutical agents against this debilitating neurological disease.

## Introduction

Genetic mutations often affect the structure of the encoded protein, resulting in structural disruption and, in some cases, disease. For example, sickle cell disease is caused by a single point mutation in the sixth codon of the β-globin gene^1–3^. A change from glutamic acid to valine induces a structural change in red blood cells that consequently adopt a sickle-like shape, and their physiological function is compromised. Similarly, a polymorphism in apolipoprotein E (APOE), a major cholesterol carrier, also demonstrates a cause-and-effect relationship between genetic risk and disease. APOE4, unlike other APOE alleles, has an arginine at residue 112, and the structure of APOE4 is more stable than other isoforms due to the formation of an additional salt bridge involving this residue^4, 5^. This structural feature of APOE4 affects its ability to bind lipids or β-amyloids (Aβ), and contributes to Alzheimer’s disease pathogenesis in an Aβ-dependent manner, although the exact mechanism is not completely understood^6^.

Kohlschutter-Tönz syndrome (KTS) is a rare genetic disorder characterized by severe global developmental delay, epilepsy, amelogenesis imperfecta, psychomotor delay or regression starting early in childhood, and intellectual disability^7–12^. Amelogenesis imperfecta, the most striking feature of KTS, is used as a clinical marker, which is most obviously observed as yellowed teeth and abnormal enamel^7^. The molecular cause of KTS has not yet been elucidated. However, recent genetic studies using a combination of whole-exome sequencing, autozygosity mapping, linkage analysis, and Sanger sequencing revealed that KTS is caused by putative loss-of-function mutations in the *ROGDI* gene on chromosome 16p13.3^13–16^. Mutations causing KTS include the premature termination of translation of Rogdi, a homozygous frameshift deletion, and an abnormal splicing error.

Rogdi is the human homolog of *Drosophila melanogaster* rogdi, and is a predicted leucine zipper protein of unknown function^13^. The protein is present in metazoan species ranging from worms to humans, and is highly conserved across taxa, indicating an essential functional role that is evolutionarily conserved. A previous study revealed that Rogdi is widely expressed, with particularly high expression in the adult brain, spinal cord, peripheral blood, heart, and bone marrow, suggesting an involvement in neurogenesis^13^. This is supported by data showing that Rogdi directly interacts with DISC1, which is necessary for neuronal proliferation, migration in cerebral interneurons, and their proper differentiation in the cerebral cortex^13, 17, 18^. Another study indicates an important role in tumorigenesis and the cell death mechanism in cervical cancer cells, in which knockdown of the *ROGDI* gene leads to increased p53 and p21 protein levels and downregulation of CDK1 and CDK2^19, 20^.

Although the genetic causes of *ROGDI* mutants in KTS patients are relatively well established, the molecular basis by which specific mutations abolish protein function and cause KTS has not yet been elucidated. Herein, we report the atomic resolution structure of the human Rogdi protein for the first time. Combined with biochemical data, the structure provides insight into the link between Rogdi mutants and KTS at the molecular level.

## Results

### Crystal Structure Determination

**Figure 1:**
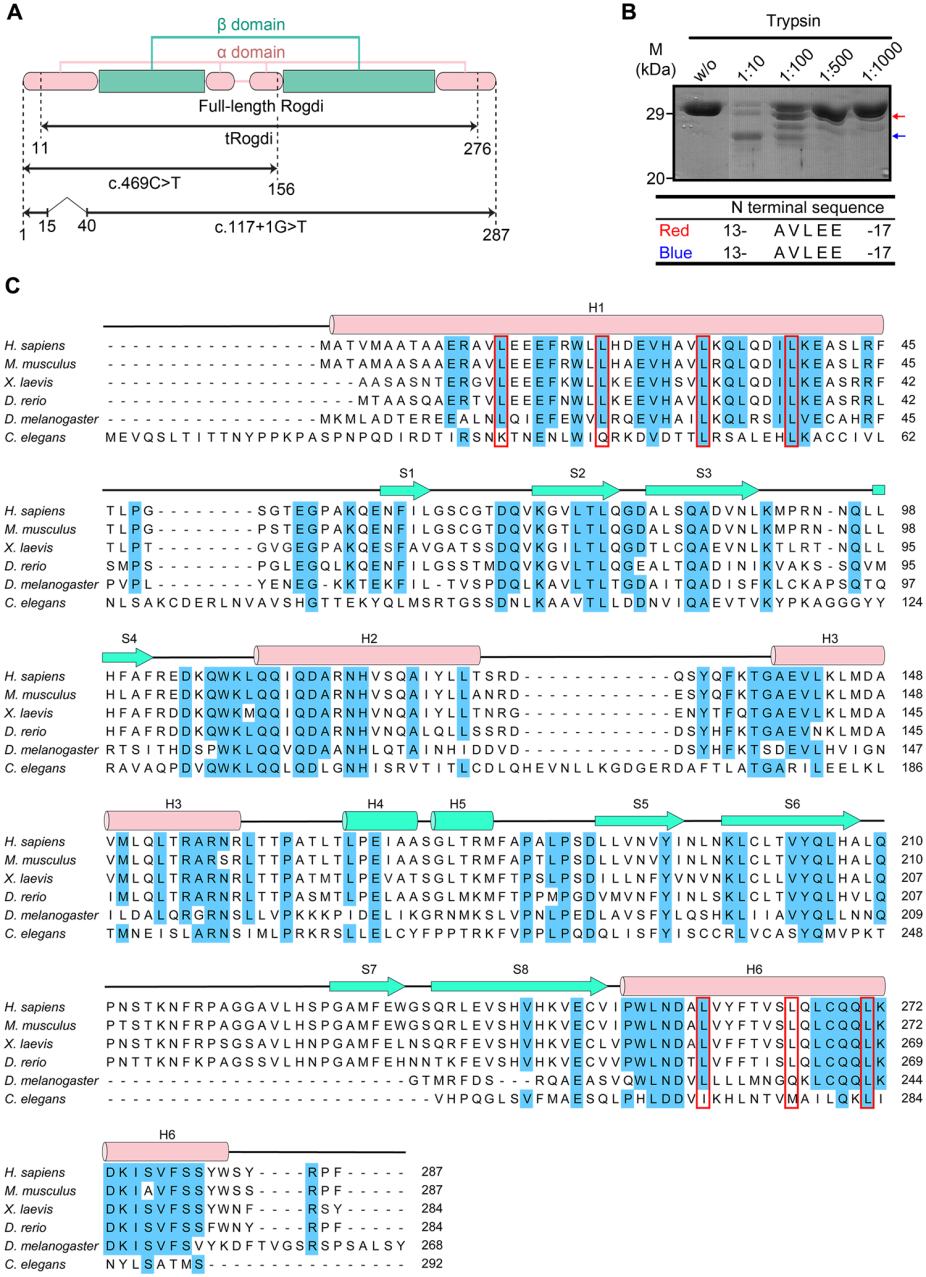
Domain Structure of Rogdi. (**A**) Diagram showing the domain structure of human Rogdi as defined by structural analysis. The crystal structures of full-length and truncated Rogdi (tRogdi, residues 11–276) were determined in this study. Two representative mutants causing KTS are shown to compare the domain structure of wild-type and mutant proteins. (**B**) Protease digestion of full-length Rogdi by trypsin. Rogdi was mixed with trypsin at the indicated trypsin:Rogdi ratio for 1 h at 4 °C. Reactions were stopped by adding SDS sample buffer, and the products were separated by SDS-PAGE. The two resistant fragments, indicated by red and blue arrows, were subjected to N-terminal sequencing. (**C**) Sequence alignment of Rogdi from six species (*H*. *sapiens*, *M*. *musculus*, *X*. *laevis*, *D*. *rerio*, *D*. *melanogaster*, and *C*. *elegans*). Secondary structural elements are indicated above the sequences, with helices depicted as cylinders and strands as arrows. Conserved residues (five out of six organisms) are highlighted in blue. The repeated heptad leucine residues in H1 and H6, which were predicted to form a leucine zipper motif, are indicated by a red box.

The human Rogdi protein comprises 287 residues, and is highly conserved among species including fish, fly, frog, worm, and mouse (Fig. 1). To gain structural insight into Rogdi mutations and their link to KTS, we crystallized human Rogdi. Native crystals of the full-length protein grew in space group R3 and diffracted to 2.8 Å resolution using a synchrotron radiation source. However, we were unsuccessful in producing full-length selenomethionine (Se-Met) derivatized protein, due to poor protein expression in the methionine auxotrophic bacteria. Instead, Se-Met substituted Rogdi was produced and crystallized using N- and C-terminally truncated constructs (residues 11–276, referred to as tRogdi). The structure of tRogdi was determined by single-wavelength anomalous dispersion (SAD) and refined to 2.04 Å resolution. The structure of full-length Rogdi was determined by molecular replacement using the tRogdi structure as a search model and refined to 2.8 Å resolution, with *R*_work_/*R*_free_ values of 21.9/26.8% (Fig. S1). Statistics for data collection and refinement are presented in Table 1.

**Table 1:** Data Collection and Refinement Statistics.

	Full-length Rogdi	tRogdi
Data set:	Native	Se-SAD
X-ray source	Beamline 5C, PAL	Beamline 5C, PAL
Temperature (K)	100	100
Space group:	R3	P2_1_2_1_2
Cell parameters a, b, c (Å)	169.00, 169.00, 220.62	63.36, 114.69, 44.21
	90.00, 90.00, 120.00	90.00, 90.00, 90.00
**Data processing**		
Wavelength (Å)	1.00000	0.9794
Resolution (Å)	50-2.80	50-2.04
R_merge_ (%)^a^	6.7 (55.1)*	8.2 (46.9)
I/σ	44.6 (5.7)	49.8 (5.9)
Completeness (%)	99.8 (100.0)	99.9 (99.9)
Redundancy	9.1 (9.2)	9.4 (9.3)
Measured reflections	273212	199950
Unique reflections	30008	21207
**Refinement statistics**		
Data range (Å)	36.17-2.80	34.57-2.04
Reflections	57748	39509
Nonhydrogen atoms	8459	1707
Water molecules	104	170
R.m.s. ∆ bonds (Å)^b^	0.006	0.004
R.m.s. ∆ angles (°)^b^	1.239	0.79
R-factor (%)^c^	21.9	20.1
R_free_ (%)^c,d^	26.8	24.5
Ramachandran plot, residues in		
Most favored regions (%)	90.5	94.4
Additional allowed regions (%)	8.6	5.1
Generously allowed regions (%)	0.8	0.0
Disallowed regions (%)	0.1	0.5

*Highest resolution shell is shown in parenthesis.

^a^R_merge_ = 100 × Σ_h_Σ_i_|*I*_i_(h) − <*I*(h)> |Σ_h_ <*I*(h)>, where *I*_i_ (h) is the *i*th measurement and <*I*(h)> is the weighted mean of all measurement of *I*(h) for Miller indices h.

^b^Root-mean-squared deviation (r.m.s. ∆) from target geometries.

^c^R-factor = 100 × Σ|F_P_ − F_P(calc)_|/Σ F_P_.

^d^R_free_ was calculated with 5% of the data.

The structures of full-length and truncated Rogdi are almost identical, with a root mean square deviation (RMSD) of 0.8 Å for all Cα atoms. Therefore, we discuss only the full-length Rogdi structure. Crystals of full-length Rogdi contain four molecules in the asymmetric unit. Interestingly, there are two conformations among the four molecules, with structural differences mainly at the N-terminus (residues 1–14), presumably due to crystal packing. The electron density at the N-terminal region is relatively well ordered in both conformations. Residues 1–14 in the two molecules fold into an α-helix (designated as Rogdi^H^), extending the α-helix to residue 45, while the same regions in the remaining two molecules are unfolded and form a long loop (designated as Rogdi^L^), suggesting that this region might be flexible in solution. Consistent with this observation, the N-terminus of Rogdi is vulnerable to proteolytic degradation (Fig. 1B). The structure of the two Rogdi^H^ molecules and the two Rogdi^L^ molecules are almost identical, with RMSD values of 0.4 and 0.2 Å for all Cα atoms. The three loop regions (residues 64–68, 92–96, and 212–221) are disordered in the two Rogdi^H^ molecules; however, the electron density for Rogdi^L^ is relatively well ordered throughout the protein chain. Apart from in these regions, no structural differences between Rogdi^H^ and Rogdi^L^ were apparent, as reflected by the RMSD values of 1.5 Å for 236 out of 255 aligned Cα atoms. The N-terminal amino acids 48–55, which correspond to the direct linker between H1 and S1 in all molecules including tRogdi, Rogdi^H^, and Rogdi^L^, were not visible in the electron density map, indicating high flexibility.

### Structure of Human Rogdi

**Figure 2:**
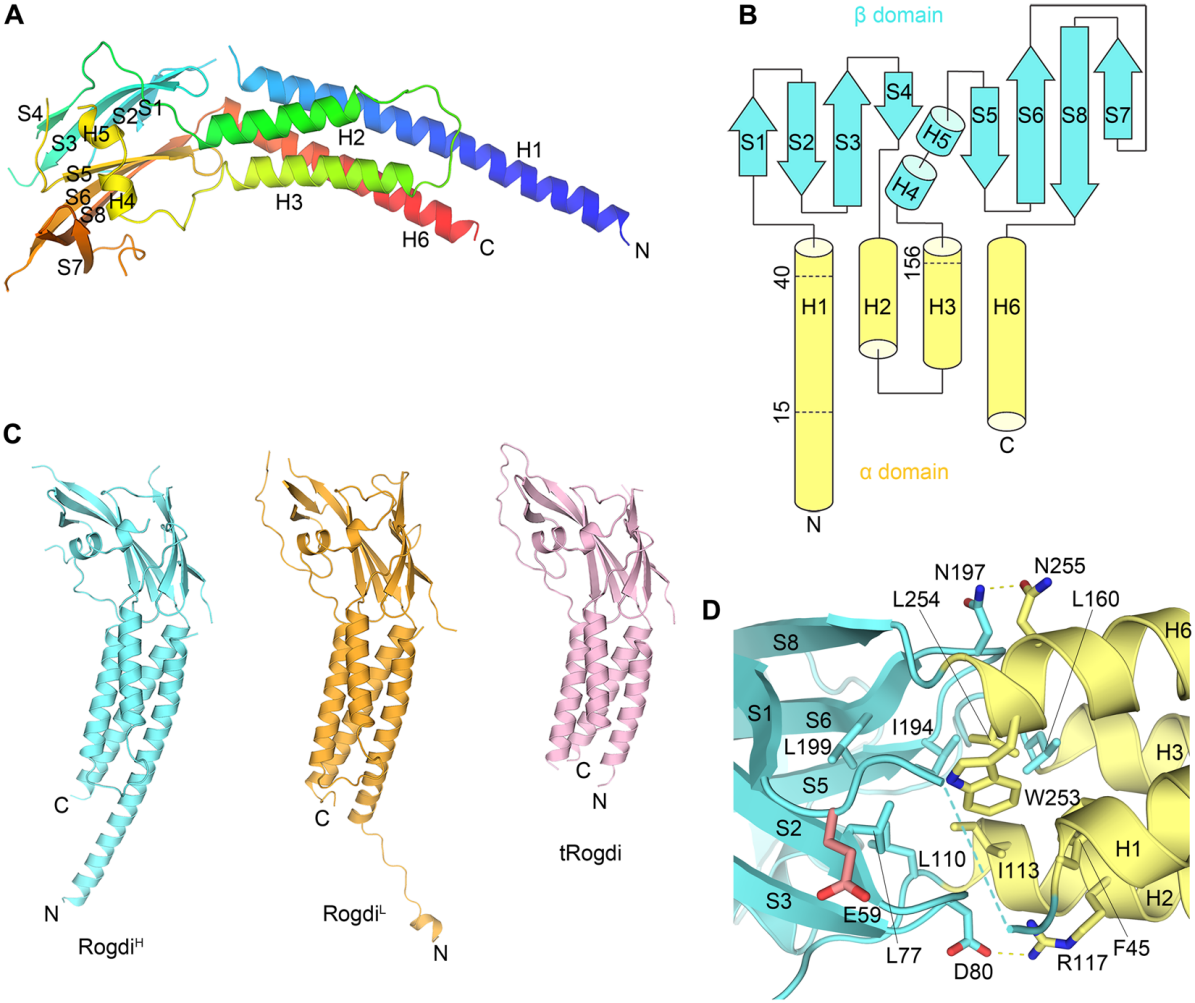
Crystal Structure of Rogdi. (**A**) Ribbon diagram showing the structure of full-length Rogdi. The protein is composed of two distinct α and β domains. The protein chain is colored blue to red from the N- to C-terminus. (**B**) Topological diagram showing the secondary structural elements and domain organization of human Rogdi. Helices and strands are depicted by cylinders and arrows, respectively. (**C**) Overall structure of full-length Rogdi (Rogdi^H^ = cyan, Rogdi^L^ = orange) and tRogdi (pink) in the same orientation for comparative purposes. (**D**) Close-up view showing residues that contribute to the tight association at the interface between the β (cyan) and β (yellow) domains. Oxygen and nitrogen atoms are colored red and blue, respectively. Yellow dotted lines indicate intermolecular hydrogen bonds. The Glu 59 residue known as a polymorphism (E59K) in human Rogdi is highlighted in red.

Molecular models of Rogdi are presented in Fig. 2 and S1. The crystal structure shows that Rogdi resembles a boomerang with an elongated curved structure comprising two distinct α and β domains. The α domain consists of four helices (H1 to H3, and H6) that form a left-handed four-helix bundle through van der Waals interactions. Overall, the α domain is a compact rod of 75 Å in length and 25 Å in diameter. The four helices lie in an anti-parallel orientation, with their axes almost parallel to each other, and a hydrophobic core lies buried within. In the four-helix bundle, H2 and H3 are connected to each other via a relatively short loop (residues 129–139), forming a helix-loop-helix structure. By contrast, the secondary structural elements between H1 and H2 and between H3 and H6 are not directly linked by short loops, and are instead separated by a β domain. However, the α domain is not completely isolated from the β domain in terms of the primary structure of Rogdi. Although the H1 helix starts from the N-terminus (residue 1), the first α-helix involved in forming the four-helix bundle starts from residue 19. Residues 1–18 of helix H1 are not directly involved in forming the four-helix bundle structure, consistent with a high degree of flexibility for this region, as indicated in the crystal structure and by the limited proteolysis results. The H1 helix of the four-helix bundle starts at residue 19 and ends at residue 45, and its adjacent anti-parallel paired helix (H6) begins from residue 252 and ends at the C-terminus (residue 287). Therefore, the α domain spans from the N-terminus to the C-terminal of the Rogdi sequence (Fig. 1A and 2B).

The β domain adopts a β-sandwich structure comprising two β-sheets that are stacked layer in a layer and twisted anticlockwise relative to each other. Both β-sheets contain four (S1 to S4 and S5 to S8) anti-parallel β-strands, and are flanked by two short α-helices (H4 and H5). The first sheet forms a canonical up-and-down anti-parallel β-strand structure. The second β-sheet folds into a more unique structure by combining a β-hairpin structure consisting of S5 and S6 with a Greek key-like motif comprising S6 to S8 plus a flexible loop (residues 209 to 228)^21^. Overall, the β domain has eight anti-parallel β-strands and two α-helices, which are packed into a compact domain with dimensions of 40 Å × 28 Å × 33 Å.

The α and β domains are not separated from each other, but rather are tightly packed together. In particular, the β-hairpin structures of each sheet are embedded in a crevice formed by one end of the two anti-parallel α-helices. To form the tight association, Leu 77 and Ile 194 from the β-strands are inserted into the center of the interface and form hydrophobic interactions with Phe 45 of helix H1, Leu 110 and Ile 113 of helix H2, Leu 160 of helix H3, and Trp 253 and Leu 254 of helix H6 from the α domain. These interacting residues are highly conserved, suggesting that the van der Waals contacts between α and β domains are preserved among different species. In addition to hydrophobic interactions, the side chains of Asp 80 and Asn 197 in the β-hairpin engage in hydrogen bonds with the side chains of residues Arg 117 and Asn 255 from the α domain helices (Fig. 2D).

According to the UniProtKB/Swiss-Prot report (ID: Q9GZN7), one polymorphism (E59K, p.Glu59Lys) has been identified in human Rogdi. We investigated the position of this residue in our crystal structure, and found that it (Glu59) is located at the end of the loop generated between H1 helix and β1 strand (Fig. 1C and 2D). The side chain of Glu59 projects toward the protein surface, and is not directly involved in interactions with other residues. Furthermore, the residue is not conserved in *C*. *elegans* (Fig. 1C). Based on these considerations, we conclude that it is unlikely that the E59K polymorphism affects the overall structure or functions of Rogdi.

### The α Domain of Rogdi adopts a Leucine Zipper-Like Structure

**Figure 3:**
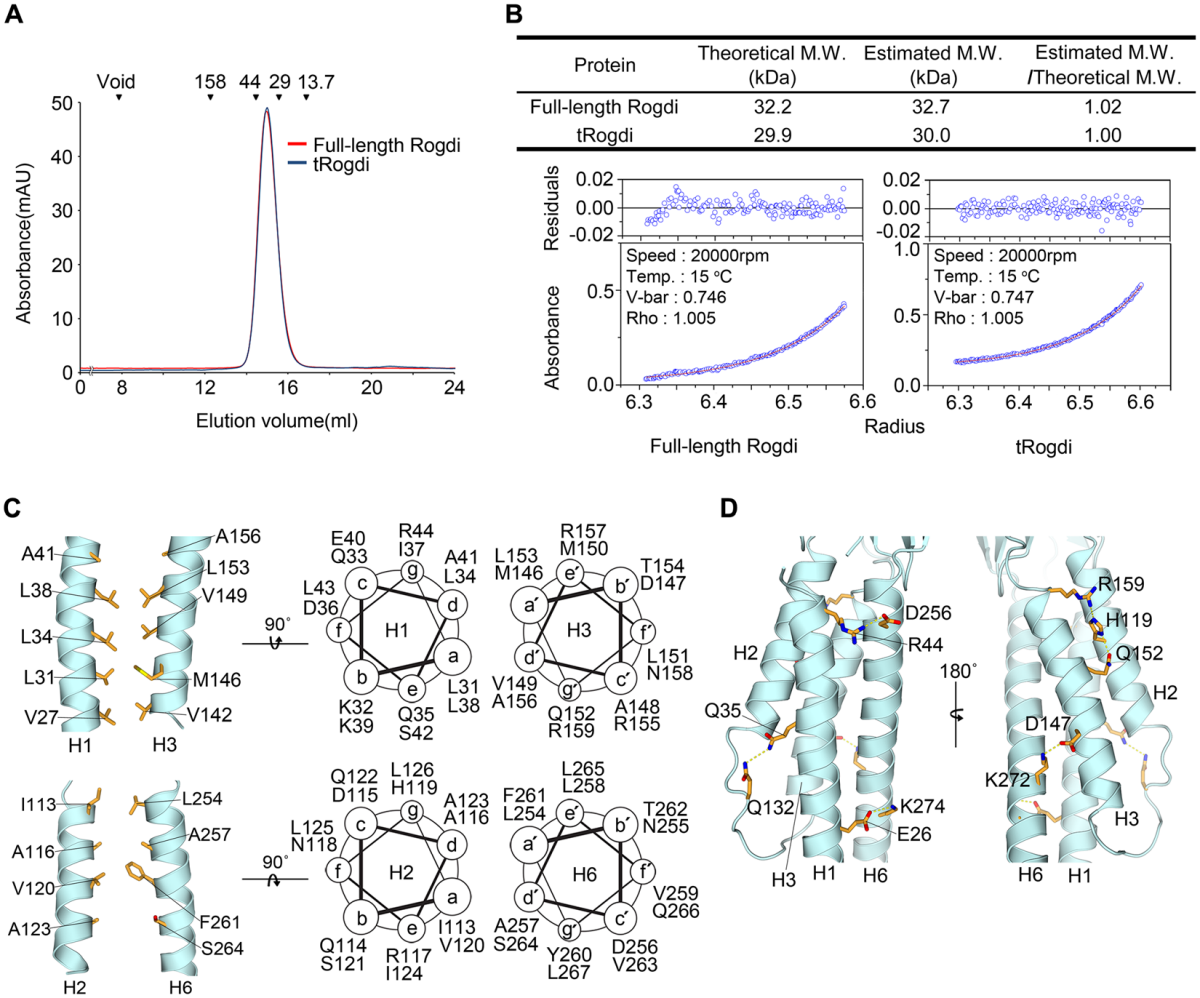
The Leucine Zipper-Like Domain of Rogdi. (**A**) Size-exclusion chromatography analysis of full-length and truncated (residues 11–276) Rogdi showing the oligomeric state. Molecular mass standards for SEC experiments (top) were aldolase (158 kDa), ovalbumin (44 kDa), carbonic anhydrase (29 kDa) and RNase A (13.7 kDa). Chromatography was performed on a Superdex 200 column in buffer containing 25 mM TRIS, 150 mM NaCl, and 5 mM DTT (pH 7.5). (**B**) Equilibrium fitting of the results of analytical ultracentrifugation for full-length (left) and truncated (right) Rogdi. The lower panel depicts the fitted overlay (red line) to the experimental data (blue circles). The upper panel depicts the residuals. (**C**) Helical wheel representation and cartoon diagrams showing the heptad repeat and intermolecular interactions within two parallel helices (H1/H3, top and H2/H6, bottom) of the Rogdi α domain. (**D**) Detailed view of the intermolecular hydrogen bonds between the surface of amphipathic helices of the α domain. Dotted lines indicate hydrogen bonds (Q35-Q132, R159-H119, and H119-Q152) and three ion pairs (see text for details).

Rogdi is a predicted leucine zipper (ZIP) protein based on sequence analysis, which reveals a periodic repetition in leucine residues at every seventh position. Most heptad leucine residues are in the N-terminal region, specifically residues 15 to 47, but residues 251 to 282 are near the C-terminus (Fig. 1C). However, the crystal structure reveals that full-length Rogdi is unexpectedly monomeric, unlike most ZIP proteins that exhibit a left-handed parallel dimeric coiled-coil structure or a higher order oligomer^22^. The monomeric organization of Rogdi was confirmed in solution by biochemical experiments including size-exclusion chromatography and analytical ultracentrifugation (Fig. 3A and B). Although Rogdi does not self-associate through the coiled-coil α domain, a set of predominantly repeated leucine residues are buried and involved in forming hydrophobic interfaces in the four-helix bundle structure. Based on this observation, we propose that two parallel helical pairs (H1/H3 or H2/H6) arrange into leucine zipper-like domains within the Rogdi monomer (Fig. 3C). The hydrophobic side chains of V27, L31, L34, and L38 of H1, and those of V142, M146, V149, and L153 of the parallel H3, are oriented towards each other, where they contribute to the stabilization of the four-helix bundle structure by forming hydrophobic interactions. Likewise, the hydrophobic side chains of I113, A116, V120, and A123 of H2, and those of L254, A257, F261, and S264 of its parallel H6, are buried at the interface and involved in hydrophobic interactions. However, the axes of H2 and H6 are not parallel, but slightly tilted. In addition to hydrophobic interactions, six hydrogen bonds are present at the surface of the amphipathic helices. In particular, the side chains of Glu 26 and Arg 44 from H1 form salt bridges with the side chains of Lys 274 and Asp 256 from H6. Furthermore, the side chains of Asp 147 and Gln 152 from H3 form H-bonds with the side chains of Lys 272 from H6 and His 119 from H2. Lastly, the side chain of Gln 35 from H1 forms an H-bond contact with the side chain of Gln 132 from loop between H2 and H3 (Fig. 3D).

**Figure 4:**
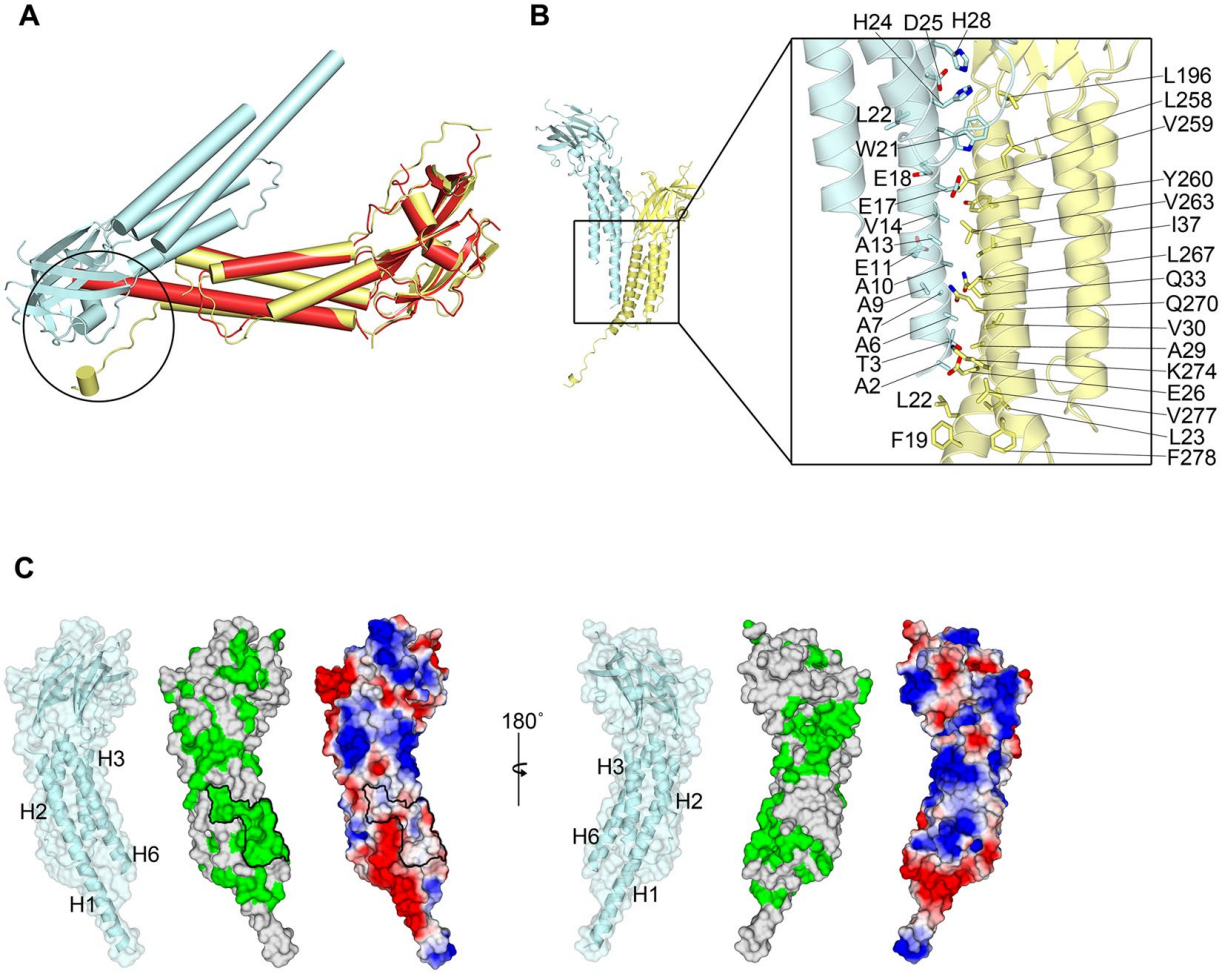
Extended H1 and H6 Helices Mediate Protein–Protein Interactions. (**A**) Crystal contacts of Rogdi^L^ in the asymmetric unit. Circles indicate the flexible N-terminal helix of Rogdi^L^, which is bent sharply due to steric hindrance. The structure of Rogdi^H^ (red) is overlaid with that of Rogdi^L^ (yellow) for structural comparison and to highlight steric hindrance between molecules in the crystal asymmetric unit. (**B**) Rogdi molecules showing crystal contacts (left). The close-up view on the right shows residues that contribute to contact surfaces. Oxygen and nitrogen atoms are colored red and blue, respectively. (**C**) Surface representation of Rogdi^H^. The surface is colored according to residue identity (middle) as shown in Fig. 1B, and according to charge distribution (right), highlighting the conservation of hydrophobic residues that might be involved in protein–protein interactions.

In general, the basic ZIP domain interacts with DNA via positively charged motifs located next to the ZIP^23^. However, the crystal structure revealed that Rogdi is likely not a DNA-binding protein, because although it has a comparable ZIP structure, it does not have the basic motif for protein-DNA interactions (Fig. 4C). Indirect evidence for DNA binding was from a previous immunostaining study using Rogdi-transfected HEK293 cells, which revealed that Rogdi is localized at the nuclear envelope^13^.

In summary, although Rogdi itself does not self-oligomerize, the α domain adopts a leucine zipper-like structure in which repeated hydrophobic residues are involved in forming the hydrophobic core of the four-helix bundle. Nevertheless, at this point, we cannot exclude the possibility that Rogdi might interact with other proteins through the coiled-coil motif of the α domain.

### Extended H1 and H6 Helices Mediate Coiled-Coil Protein Interactions

As mentioned earlier, crystals of full-length Rogdi revealed two conformations, designated as Rogdi^H^ and Rogdi^L^, depending on the continuity of H1 helix. Given that the N-terminal residues 1–11 are easily accessible and prone to proteolytic degradation, the non-paired region of H1 in the four-helix bundle (residues 1–14) might be flexible in solution. This is consistent with the structure of Rogdi^L^, in which H1 helix residues 1–14 are disassociated from the helix and form a sharp bend due to steric hindrance by neighboring molecules in the crystal (Fig. 4A). However, this region might undergo a conformational change and adopt a helical structure with a parallel helix from a neighboring molecule, as shown in Rogdi^H^ (Fig. 4B). The extended H1 helix in Rogdi^H^ engages in a parallel coiled-coil interaction with the adjacent H1 and H6 helices from the other molecule via crystal packing. The interface residues facing the coiled-coil domain are composed of hydrophobic residues such as Ala 6, Ala 10, Val 14 and Trp 21 from H1, and Val 259, Tyr 260, Val 263, and Leu 267 from H6. In particular, a highly conserved hydrophobic patch comprising Leu 23, Ile 37, Leu 267, and Val 277 from H1 and H6 is exposed to the surface, suggesting that the hydrophobic surface generated by helices H1 and H6 could provide a platform for a protein–protein interaction module by conformational changes of the N-terminus into an extended H1 helix (Fig. 4C).

Previous studies using the yeast-two hybrid technique discovered that Rogdi might interact with DISC1 (MIM 605210), a protein implicated in the development of schizophrenia and involved in cytoskeletal stability and organization, neuronal migration, intracellular transport, and cell division^13, 17, 18^. Future work will be required to address whether the extended H1 helix might be involved in interactions with other binding partners such as DISC1.

### Rogdi Resembles Claudin-15, a major Tight Junctions Component

**Figure 5:**
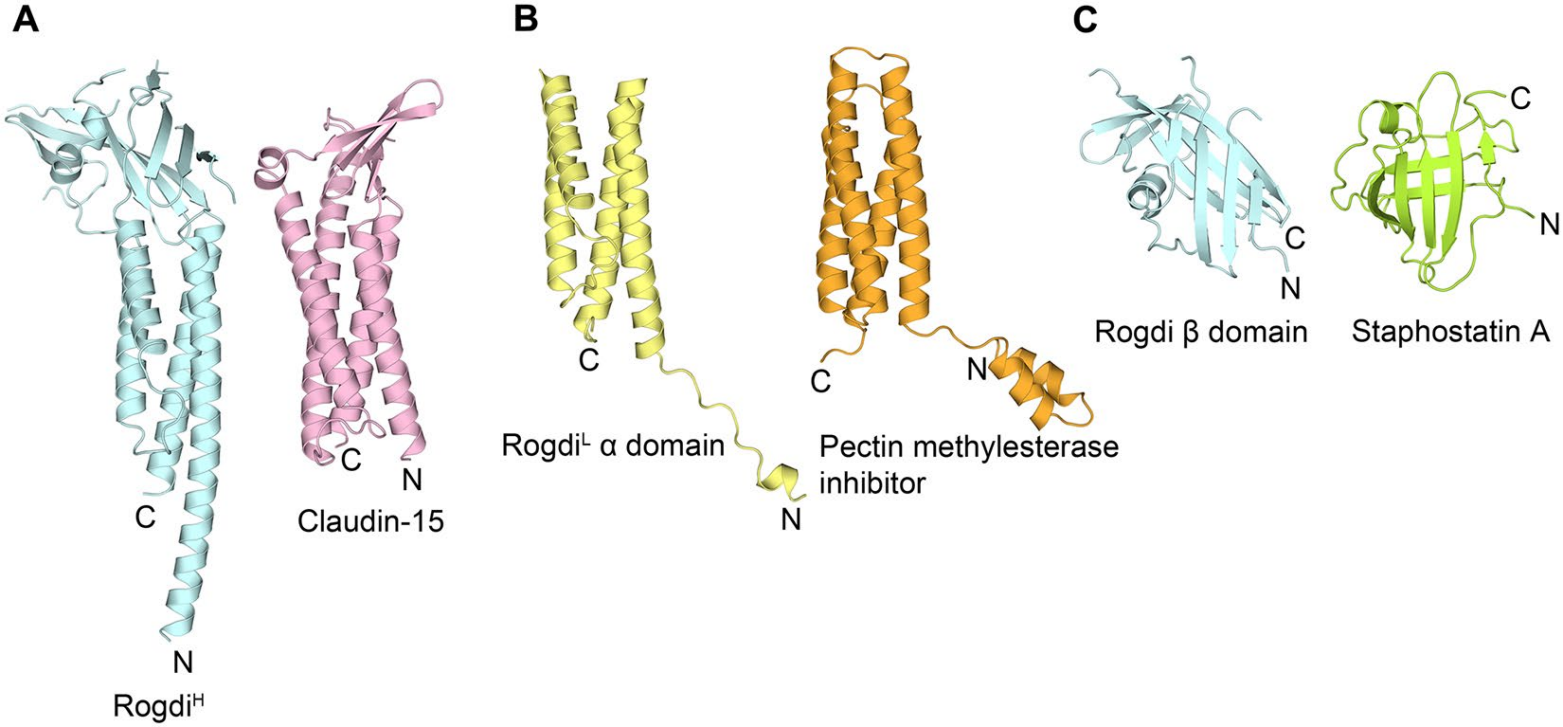
Structural Comparison between Rogdi and Claudin-15. Structural comparison of Rogdi and proteins with a similar fold, shown in the same orientation. Full-length Rogdi^H^ most closely resembles Claudin-15 (**A**, PDB ID: 4P79), an essential component of tight junctions. Rogdi^L^ shows structural similarity with pectin methylesterase inhibitor (**B**, PDB ID: 1X8Z) and staphostatin A (**C**, PDB ID: 1OH1).

To obtain insight into the molecular functions of Rogdi based on its structure, we searched the Protein Data Bank (PDB) using DALI^24^, and found that full-length Rogdi is most similar to Claudin-15 (Z-score = 8.4), which is a major membrane component of tight junctions^25^. In particular, the structure of Rogdi appears to share four structural features with Claudin-15 (Fig. 5A). (i) Claudin-15 is also composed of a characteristic β-sheet fold and a four-helix bundle; (ii) the typical left-handed four-helix bundle of Claudin-15 spans the entire protein chain from the N- to the C-terminus, as is the case for the α domain of Rogdi; (iii) the β-strands in the β-sheet are organized in an anti-parallel manner and are flanked by a short α-helix; and (iv) the two distinct α and β domains are not spatially separated but tightly packed together. Since these structural features are also shared with the Rogdi structure, the two proteins could conceivably share similar functions, most likely as scaffolds and/or engaging in self-polymerization in tight junctions^25^. Other than Claudin-15, the full-length Rogdi structure did not share a great deal of similarity with any other soluble proteins.

Although the full-length Rogdi structure could only be aligned with Claudin-15, the separate domains could be superimposed with pectin methylesterase inhibitor and staphostatin A with an RMSD of 3.3 and 5.7 Å, respectively (Fig. 5B and C). However, we propose that the similarity with these domains does not clearly reflect their molecular functions, because the RMSD values do not indicate a close structural similarity, and the two distinct domains of Rogdi are not separate but are tightly associated with each other.

### The Relationship between Rogdi Mutations and KTS

**Figure 6:**
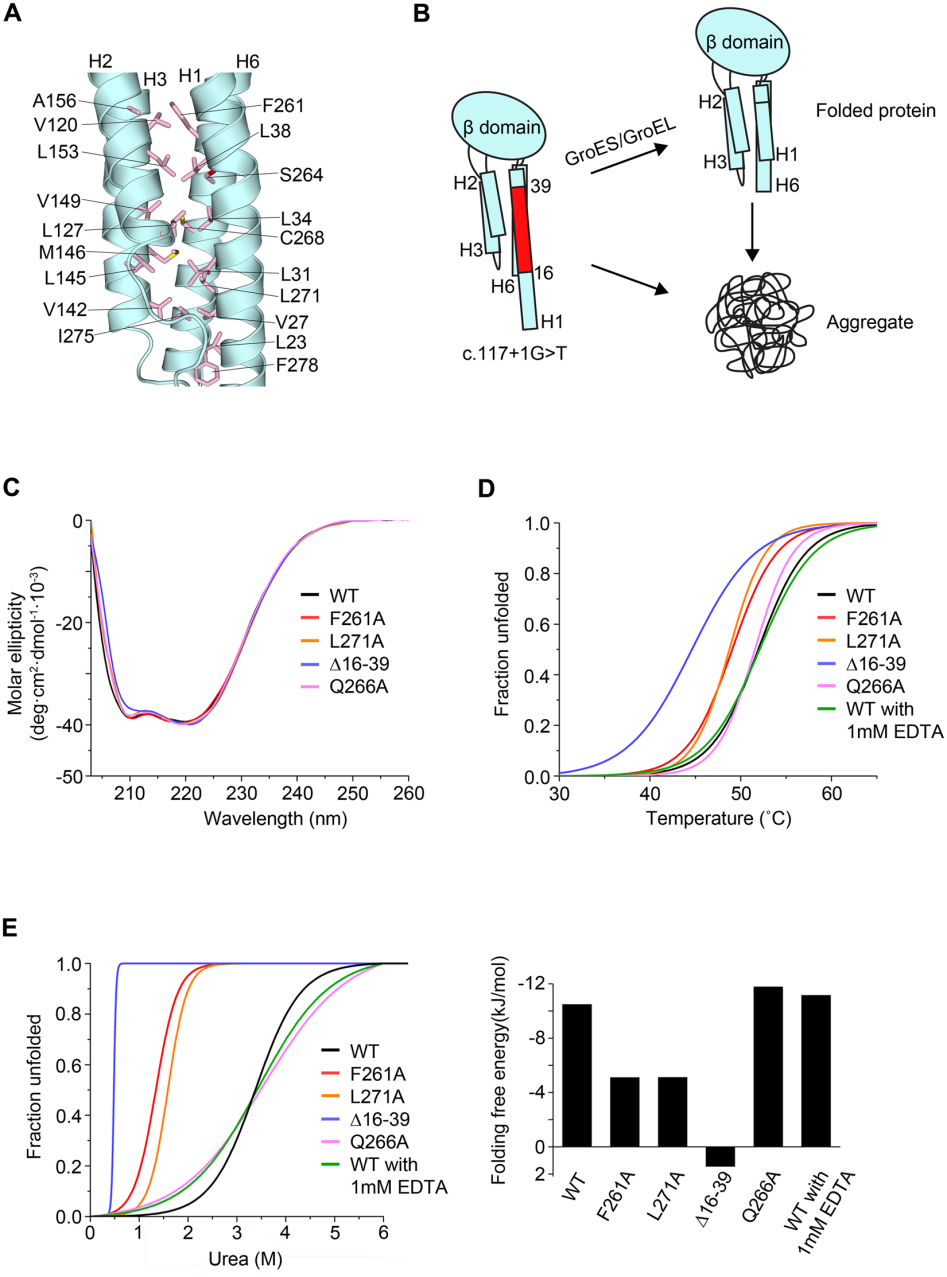
Mutation of Rogdi affects Structure and Stability. (**A**) Close-up view of the four-helix bundle hydrophobic interface in the α domain of Rogdi. (**B**) Schematic diagram showing the possible domain organization in the c. 117 + 1 G > T mutant^15^. The red region (residues 16–39) of H1 is deleted in the mutant due to a splicing error. (**C**) Far-UV CD spectra of wild-type and mutant Rogdi scanned from 203 to 260 nm. (**D**) Thermal unfolding curves of wild-type and mutant Rogdi measured by CD spectroscopy. (**E**) Urea-induced unfolding of wild-type and mutant Rogdi^39^ conducted by monitoring loss of helicity during CD spectroscopy at 222 nm.

Extensive genetic analyses have revealed that mutations in the *ROGDI* gene appear to cause KTS. Mutations of Rogdi implicated in KTS are summarized in Table 2. All mutations identified to date are frameshift, nonsense, or splice-site mutations that are expected to either cause premature mRNA degradation by nonsense-mediated decay, or dramatically alter the protein structure and thereby cause a complete loss of protein function. In particular, *ROGDI* c.469 C > T and c.286 C > T mutations cause the premature termination of translation at residue 157 and residue 96, respectively^14^. Residue 157 is located at the C-terminal end of H3, indicating that this mutant is truncated at the second β-sheet and H6. Since H6 is a component of the four-helix bundle in the α domain, the mutant would disrupt both α and β domains (Fig. 1A and 6A). Recently, the novel *ROGDI* homozygous mutation (c.117 + 1 G > T) was identified in a patient with KTS^15^. The mutation abolishes the usual splice donor site of intron 2, which causes the deletion of exon 2 and the in-frame assembly of exon 3. Exon 2 encodes a highly conserved 24 amino acid region (residues 16–39), which corresponds to the central region of H1 (Fig. 1A). Apart from these residues, the mutant protein would have no further truncations. Deletion mutations may reduce the length of helix H1 from 45 to 20 amino acids, or disrupt the structure of the H1 helix itself. In both cases, mutation would break the hydrophobic core responsible for stabilizing the four-helix bundle (Fig. 6B). Based on this hypothesis, we propose that defects in the α domain would affect the stability and hence the molecular function of the Rogdi protein, and thereby cause KTS. Notably, there are numerous reports demonstrating that the stability of four-helix bundles is essential for the structure and function of proteins^26–28^.

**Table 2:** Summary of KTS-associated *ROGDI* gene mutations.

Type of Mutation	Genotype	Molecular Phenotype	Ref.
Deletion mutation	homozygous for c.229_230del	p.Leu77Alafs*64	14
	homozygous for c.507delC	p.Glu170Argfs*72	16
	homozygous for c.46–37_46–30del	N/A	16
	homozygous for c.45 + 9_45 + 20del	p.Glu16Valfs*57	16
Nonsense mutation	homozygous for c.286 C > T	p.Gln96*	14
	homozygous for c.469 C > T	p.Arg157*	13
Duplication and deletion mutation	heterozygous for c.366dupA and c.45 + 9_45 + 20del	p.Ala123Serfs*19	16
Splicing-site mutation	heterozygous for c.531 + 5 G > C and c.532-2 A > T	N/A	14
	homozygous for c.117 + 1 G > T	24A.A. deletion from position Glu16 to Lys39	15

To further investigate the potential importance of the α domain to the stability of the Rogdi protein, we generated point mutant constructs (F261A and L271A) to induce interruptions in the hydrophobic core of the α domain, and measured protein stability using circular dichroism (CD) spectroscopy. A wavelength scan of the mutants showed no significant differences in spectra, suggesting that mutations did not affect the secondary structure (Fig. 6C). We determined the folding free energy value of wild-type and mutant Rogdi proteins using chemical and thermal denaturation experiments in which the loss of α-helical structure was monitored by CD spectroscopy at neutral pH. Surprisingly, the melting temperatures for the F261A and L271A mutants were decreased by ~4–5 °C compared with the wild-type protein and a negative control mutant (Q266A), suggesting the disruption of the hydrophobic core destabilizes the helical bundle of the α domain and affects the overall protein stability (Fig. 6D). Consistent with the thermal stability data, all affected mutants exhibited significantly less resistance to urea-induced protein denaturation (Fig. 6E).

Most interestingly, although deletion of the N-terminus (residues 16–39) did not affect the CD wavelength spectrum, the dramatic disruption of the thermal and chemical stability indicated that the deletion affected the protein structure and stability, which might explain the KTS disease-causing phenotype (Fig. 6B–E). It is still unknown whether the α domain is involved in protein–protein interactions, and what the molecular functions of such interactions might be. However, we believe that the α domain of Rogdi contributes to the overall structure and stability, and perturbation of the α domain by mutation likely results in the loss of function and the eventual onset of KTS.

## Discussion

Rogdi has been predicted to be a leucine zipper protein and a transcription factor. However, our crystal structure revealed that the N- and C- termini of Rogdi include periodically repeating leucine residues that contribute to the formation of a four-helix bundle structure rather than a typical leucine zipper, although the α domain does adopt an internal ZIP-like structure (Fig. 3C). Most importantly, we also found that the N-terminal H1 helix (residues 19–45) is paired with the C-terminal H6 helix (residues 252–287) in an anti-parallel manner, indicating that the integrity of the four-helix bundle requires both N- and C-terminal residues (Fig. 2B). Thus, truncation mutations might seriously affect the overall structure, and cause the protein to be degraded (Fig. 6). Mounting evidence suggests mutation of Rogdi is the genetic cause of KTS. In particular, quantitative RT-PCR data from KTS patients showed that transcription of Rogdi is lower than in wild-type controls, indicating that mutated transcripts are selectively degraded through a nonsense-mediated decay^14^. In the present study, we further demonstrated that even if translation of mutated Rogdi transcripts is completed, the structure of the truncated Rogdi protein is likely to be unstable and prone to degradation, based on the crystal structure. The α domain in particular appears to be very important for the overall structure and stability, and presumably for mediating protein–protein interactions.

Rogdi appears to share several key structural features with Claudin-15 (Fig. 5A). In addition, it was recently reported that Claudin-4 and Claudin-19, which are structurally similar to Claudin-15, interact with a *Clostridium perfringens* enterotoxin via their β-sheet structures, resulting in the disintegration of tight junctions^29, 30^. Although future work is clearly necessary, the β domain of Rogdi might also perform essential molecular functions, such as providing a binding platform for interacting partners.

Recently, patients showing atypical KTS phenotypes without any mutations in the *ROGDI* gene have been reported, suggesting that other genes might also cause KTS^14, 16, 31^. Indeed, according to the report, mutation in the *SLC13A5* gene, which encodes a sodium-dependent citrate transporter, might also cause KTS^32^. Moreover, the *ROGDI* gene is reported to play an important role in tumorigenesis and the cell cycle^19, 33^. However, it has not yet been elucidated whether there is a molecular correlation between *ROGDI* and *SLC13A5* in cancerous cells. Therefore, further study is needed to expand our understanding of the cellular functions of Rogdi and its relationship to KTS phenotypes. Nevertheless, the structural and biochemical results of the present study provide structural insight into the relationship between Rogdi and KTS, and this fundamental knowledge might prove useful for the development of pharmaceutical agents for the treatment or cure of this debilitating neurological disease. Furthermore, the high-resolution structure of Rogdi can be used to confirm or repudiate the potential effects of new and potentially pathogenic mutations on Rogdi structure.

## Methods

### Protein Production

Human full-length (residues 1–287) and truncated (tRogdi, residues 11–276) Rogdi proteins were expressed in *E*. *coli* BL21(DE3) cells from a modified pET-Duet vector with an N-terminal His_6_ tag followed by a TEV protease cleavage site. Cells were lysed in 25 mM sodium phosphate (pH 7.5) containing 400 mM NaCl and protease inhibitors. After Ni^2+^ affinity chromatography, the His_6_ tag was cleaved by TEV protease, and the protein was purified further by ion-exchange and gel-filtration chromatography steps. Selenomethionine-substituted protein was generated by expressing the tRogdi protein in *E*. *coli* B834 (DE3) cells (Novagen) using M9 minimal medium plus selenomethionine. The protein was concentrated to ~20 mg/ml by centrifugation in 25 mM TRIS-HCl containing 150 mM NaCl and 5 mM DTT (pH 7.5) and flash-frozen in liquid nitrogen for storage. Rogdi mutants were generated using PCR-based methods. All mutants were overexpressed at room temperature in the same *E*. *coli* host as glutathione S-transferase (GST) fusion proteins using the pGEX-6P1 vector (GE Healthcare). To increase the solubility of the deletion mutant, we co-expressed with bacterial GroESL chaperone proteins. The GST-Rogdi mutants were purified using glutathione S-sepharose and cleaved by PreScission protease (GE healthcare). Mutant proteins were purified further by cation-exchange chromatography on a HiTrap SP column and by gel-filtration chromatography on a Superdex 200 column.

### Crystallization and Structure Determination by SAD

Human tRogdi protein was crystallized at 4 °C by the hanging-drop vapor diffusion method by adding 1 μl of a 9 mg/ml protein solution to 1 μl of well solution comprising 11% PEG 4 K, 100 mM MES pH 6.5, and 5 mM DTT. The resultant orthogonal crystals belong to space group P2_1_2_1_2 (a = 63.4 Å, b = 114.7 Å, c = 44.2 Å) and contain one molecule in the asymmetric unit. For X-ray diffraction experiments, crystals were transferred to well solution containing 30% glycerol and flash-frozen in liquid nitrogen. Single-wavelength anomalous diffraction (SAD) data were collected from a Se-Met crystal at beamline 5 C of the Pohang Accelerator Laboratory (PAL) and processed using HKL-2000 software^34^. SAD data analysis was performed using Phenix^35^ software using data between 50 and 2.04 Å resolution. Phenix found eight of the nine selenium sites and refined them to a mean figure-of-merit of 0.38. Electron density modification using RESOLVE software^36^ yielded an initial electron density map of excellent quality. Successive rounds of model building and refinement were performed using Coot and Phenix, respectively^35, 37^. The final model contains one tRogdi monomer in the asymmetric unit. Residues 11 to 23, 47 to 56, 64 to 68, 92 to 96, 130 to 138, and 266 were not modelled due to weak electron density.

For full-length Rogdi crystals, protein solution (1 μl) was mixed with an equal volume of 2 M sodium chloride, 130 mM magnesium chloride, and 100 mM TRIS pH 7.0. Crystals (space group R3; a = b = 169.0 Å, c = 220.6 Å) grew within 1 week at 4 °C. For diffraction experiments, crystals were transferred to well solution containing an additional 30% glycerol and flash-frozen in liquid nitrogen. X-ray diffraction data were collected at the same PAL beamline and processed as described above. The structure was solved by molecular replacement using Phaser^38^ with the tRogdi structure as the search model. An initial model of tRogdi was improved by rigid-body and positional refinement, and the structure was built into the resulting electron density map. Final refinement yielded an R-factor of 21.9% (R_free_ = 26.8%) for data between 35 and 2.8 Å resolution (Table 1). The final model consists of 8459 protein atoms and contains four copies of Rogdi in the asymmetric unit. Of these, the following residues were not modelled due to weak electron density: Residues 48 to 55, 211, 235, and 286 to 287 in the first copy; residues 48 to 55, 211, and 286 to 287 in the second copy; residues 48 to 56, 64 to 68, 92 to 96, 212 to 221, and 284 to 287 in the third copy; residues 48 to 56, 64 to 68, 94 to 96, 181, 212 to 221, and 284 to 287 in the fourth copy. Structure analyses and molecular visualization were performed using PYMOL program (www.pymol.org). The refinement statistics and composition of the final models are summarized in Table 1.

### CD Spectroscopy

The secondary structure of Rogdi mutants were monitored using a CD spectrometer (Jasco J-815) with wavelength scans from 190 to 260 nm. Possible conformational changes were also monitored by CD spectroscopy at various temperatures by raising the temperature from 25 to 85 °C over a 5 min period, with detection at 222 nm. All samples were prepared in 25 mM TRIS pH 7.5, 150 mM NaCl, 5 mM β-mercaptoethanol, pH 7.5. For chemical denaturation, protein samples were mixed with 0 to 8 M urea, incubated for 5 h at room temperature, and measured at 222 nm on the same CD spectrometer.

### Unfolding Titration for Determination of Folding Free Energy

To determine the folding free energy of Rogdi proteins, we assumed that all proteins had folded and unfolded states that were dependent on the concentration of urea, and that the two states were reversible, as summarized in the following expressions:

1 $${\rm{Unfolded}}\,{\rm{protein}}{\boldsymbol{\rightleftarrows }}{\rm{folded}}\,{\rm{protein}}$$

In the reaction (1), the folding constant, K_F_, was calculated from the following formula:

2 $${{\rm{K}}}_{{\rm{F}}}=[{\rm{F}}]/[{\rm{U}}]$$

where [F] is the concentration of the folded protein, and [U] is the concentration of the unfolded protein. Each concentration can be expressed in terms of the total protein concentration (P_t_) and the fraction unfolded (f_U_). Equation (2) can be expressed in terms of P_t_ and f_U_ as follows:

3 $$\begin{array}{rcl}{{\rm{K}}}_{{\rm{F}}} & = & {{{\rm{P}}}_{{\rm{t}}}}^{\ast }(1-{{\rm{f}}}_{{\rm{U}}})/({{\rm{P}}}_{{\rm{t}}}\ast {{\rm{f}}}_{{\rm{U}}})\\  & = & (1-{{\rm{f}}}_{{\rm{U}}}){/f}_{{\rm{U}}}\end{array}$$

The fraction unfolded is then calculated from the observed ellipticity ([*θ*]) as follows:

4 $${{\rm{f}}}_{{\rm{U}}}=([\theta ]-{[\theta ]}_{{\rm{F}}})/({[\theta ]}_{{\rm{U}}}-{[\theta ]}_{{\rm{F}}})$$

The [*θ*]_F_ is the ellipticity of the fully folded protein, and [*θ*]_U_ is the ellipticity of the fully unfolded protein. The free energy of the protein is calculated using the following formula:

5 $${\rm{\Delta }}{\rm{G}}=-{\rm{RT}}\,\mathrm{ln}({{\rm{K}}}_{{\rm{F}}})$$

where R is the gas constant and T is the absolute temperature. The free energy of the protein can then be calculated depending on the concentration of urea ([urea]). To determine the folding free energy of the protein (ΔG°), ΔG and [Urea] are fitted to a y = ax + b plot using the following:

6 $${\rm{\Delta }}{\rm{G}}={\rm{m}}[{\rm{u}}{\rm{r}}{\rm{e}}{\rm{a}}]+{\rm{\Delta }}{\rm{G}}^\circ $$

where m is the slope, and ΔG is the folding free energy when the concentration of urea is zero.

### Analytical Ultracentrifugation

The molecular mass of human full-length and truncated Rogdi was analyzed by analytical ultracentrifugation (Optima XL-A; Beckman) using the sedimentation equilibrium technique. For sedimentation equilibrium analytical ultracentrifugation, protein samples were prepared in buffer containing 25 mM TRIS-HCl (pH 7.5), 150 mM NaCl, and 5 mM β-mercaptoethanol at concentrations of 10 μM, 15 μM, and 20 μM. Data were evaluated using a nonlinear least-squares curve-fitting algorithm in the XL-A data analysis software. For equilibrium analysis, scans obtained at equilibrium using multiple speeds (10000, 20000 and 30000 rpm) were collected at 15 °C using an An-60 Ti rotor (Beckman) by measuring the absorbance at 280 nm. Measurements were fitted to a single species model using Origin 6.03 software (Beckman Coulter, Inc.).

### Sequence Analysis

For sequence alignment (Fig. 1C), Rogdi sequences from six organisms were aligned: *Homo sapiens* (NP_078865), *Mus musculus* (NP_573448), *Xenopus laevis* (AAH94137), *Caenorhabditis elegans* (NP_498641), *Danio rerio* (NP_956257), and *Drosophila melanogaster* (NP_648956).

### Limited Proteolysis

Limited proteolysis experiments were carried out to define domain boundaries. Full-length Rogdi was digested with trypsin using a range of protein concentrations and incubation times at 4 °C. The reaction was stopped with 1 mM PMSF, and products were analyzed by SDS-PAGE and N-terminal protein sequencing.

## Electronic supplementary material

Figure S1
