# Supplementary material for: The crystal structure of human Rogdi provides insight into the causes of Kohlschutter-Tönz Syndrome
Source: Sci Rep. 2017 Jun 21;7:3972. doi: 10.1038/s41598-017-04120-x (PMC5479863; doi:10.1038/s41598-017-04120-x)
Supplement: Supplementary file 1 — Figure S1 [file 41598_2017_4120_MOESM1_ESM.pdf]

The crystal structure of human Rogdi provides insight into the causes of Kohlschutter-Tönz Syndrome

Hakbong Lee[1,4], Hanbin Jeong[1,4], Joonho Choe[2], Youngsoo Jun[3,4], Chunghun Lim[1] and Changwook Lee[1,4]*

[1]Department of Biological Sciences, School of Life Sciences, Ulsan National Institute of Science and Technology, 50 UNIST-gil, Ulsan 44919, Republic of Korea

[2]Department of Biological Sciences, Korea Advanced Institute of Science and Technology, Daejeon 34141, Republic of Korea

[3]School of Life Sciences and [4]Cell Logistics Research Center, Gwangju Institute of Science and Technology, Gwangju 61005, Rep. of Korea.

*Correspondence to:

Changwook Lee, Ph.D.

Department of Biological Sciences, School of Life Sciences

Ulsan National Institute of Science and Technology (UNIST)

50 UNIST-gil, Ulsan 44919, Republic of Korea.

Telephone – 82-52-217-2534; Fax – 82-52-217-2639

E-mail – changwook@unist.ac.kr

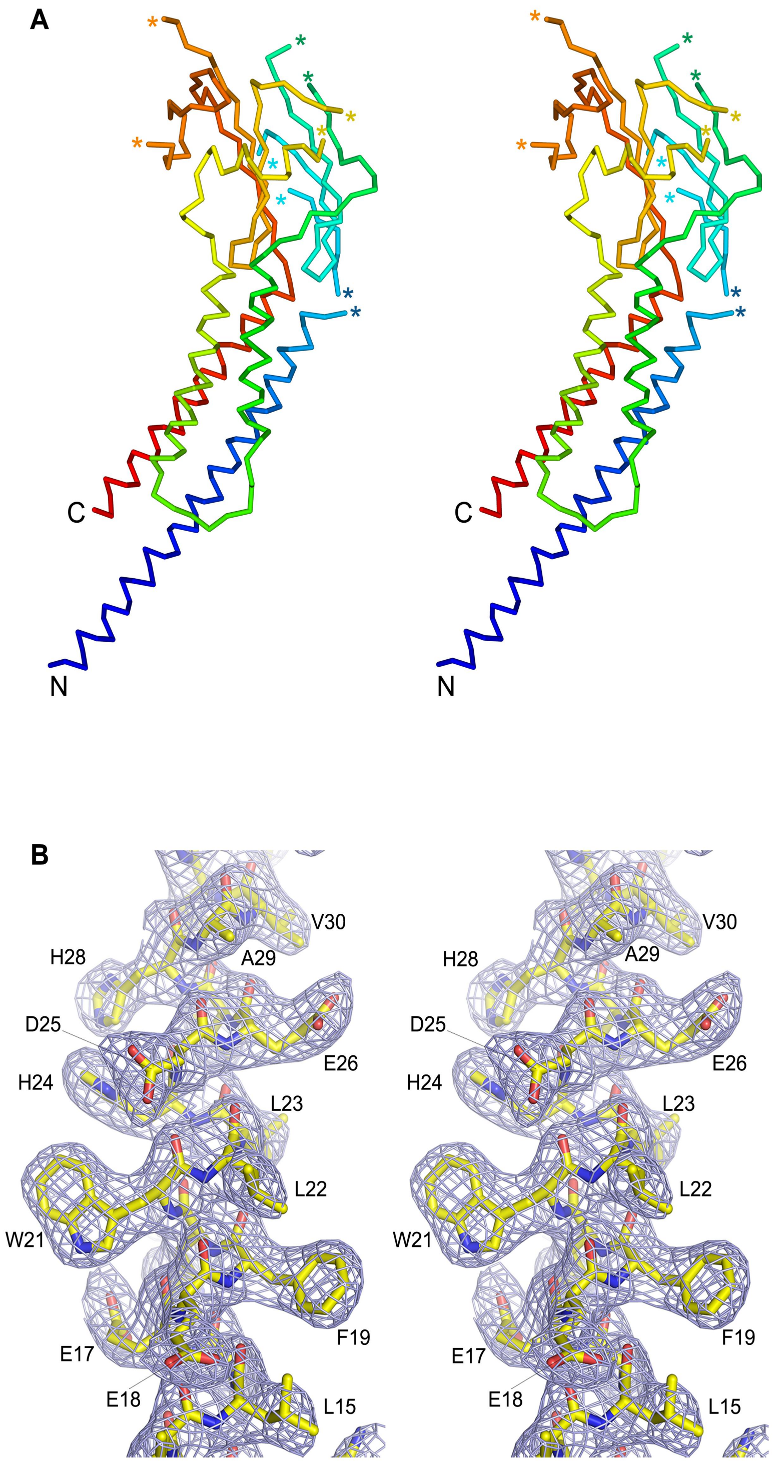

Figure S1. Stereo images of full-length Rogdi^{H}

(**A**) The stereo view shows the backbone trace of full-length Rogdi^{H}. The residues 48–56, 64–68, 94–96, 181, and 212–221 are disordered and indicated by blue, cyan green, yellow, and orange stars, respectively.

(**B**) The 2.8 Å resolution 2Fo-Fc electron density (contoured at 1σ) of the H1 in the α domain was shown with a final model to represent the quality of structural data.
